# Mortality among PCR negative admitted Ebola suspects during the 2014/15 outbreak in Conakry, Guinea: A retrospective cohort study

**DOI:** 10.1371/journal.pone.0180070

**Published:** 2017-06-30

**Authors:** Brecht Ingelbeen, Elhadj Ibrahima Bah, Tom Decroo, Idrissa Balde, Helena Nordenstedt, Johan van Griensven, Anja De Weggheleire

**Affiliations:** 1 Médecins Sans Frontières, Operational Centre Brussels, Conakry, Guinea; 2 Centre Hospitalier Universitaire de Donka, Conakry, Guinea; 3 Médecins Sans Frontières, Operational Centre Brussels, Operational Research Unit, Brussels, Belgium; 4 Department of Public Health Sciences, Karolinska Institutet, Stockholm, Sweden; 5 Clinical Sciences Department, Institute of Tropical Medicine, Antwerp, Belgium; Tulane University, UNITED STATES

## Abstract

Non-cases are suspect Ebola Virus Disease (EVD) cases testing negative by EVD RT-PCR after admission to an Ebola Treatment Centre (ETC). Admitting non-cases to an ETC prompts concerns on case- and workload in the ETC, risk for nosocomial EVD infection, and delays in diagnosis and disease-specific treatment. We retrospectively analysed characteristics, outcomes and determinants of death of EVD cases and non-cases admitted to the Conakry ETC in Guinea between 03/2014 and 09/2015. Of the 2362 admitted suspects who underwent full confirmatory PCR testing, 1540 (65.2%) were non-cases; among them 727 needed repeated confirmatory PCR testing resulting in 2.5 days (average) in the ETC isolation ward. Twenty-one patients tested positive on the repeat test, most in a period of flawed sampling for the initial test and none after introduction of PCR confirmation with geneXpert. No readmissions following nosocomial EVD infection were recorded. No combination of symptoms yielded acceptable sensitivity and specificity to allow differentiating confirmed from non-cases. Symptoms as ocular bleeding/redness have high specificity, but limited usefulness as not common. Admission delay and age distribution were not different for both groups. In total, 98 (20.6%) of 475 deaths in the ETC were non-cases. Most died within 24 hours after admission. Living in Conakry (aOR 1.78 (1.08–2.96)) was the strongest risk factor for death. Weeks with higher admission load had lower case fatality among non-cases, probably because more acute (and treatable) illnesses of contacts of known cases were admitted. These findings show high numbers of potentially critically ill non-cases need to be considered when setting up triage and referral of EVD suspect cases. Symptoms and risk factors alone do not allow differentiating the non-cases. Integration of highly-sensitive EVD diagnostic methods with short turnaround time in the triage of peripheral hospitals and dropping the systematic 2nd PCR for symptomatic early presenters could limit delays in access to adapted care of cases and seriously ill non-cases. Whether feasible without compromising outbreak control, and under which conditions, should be further assessed.

## Introduction

**Fig 1:**
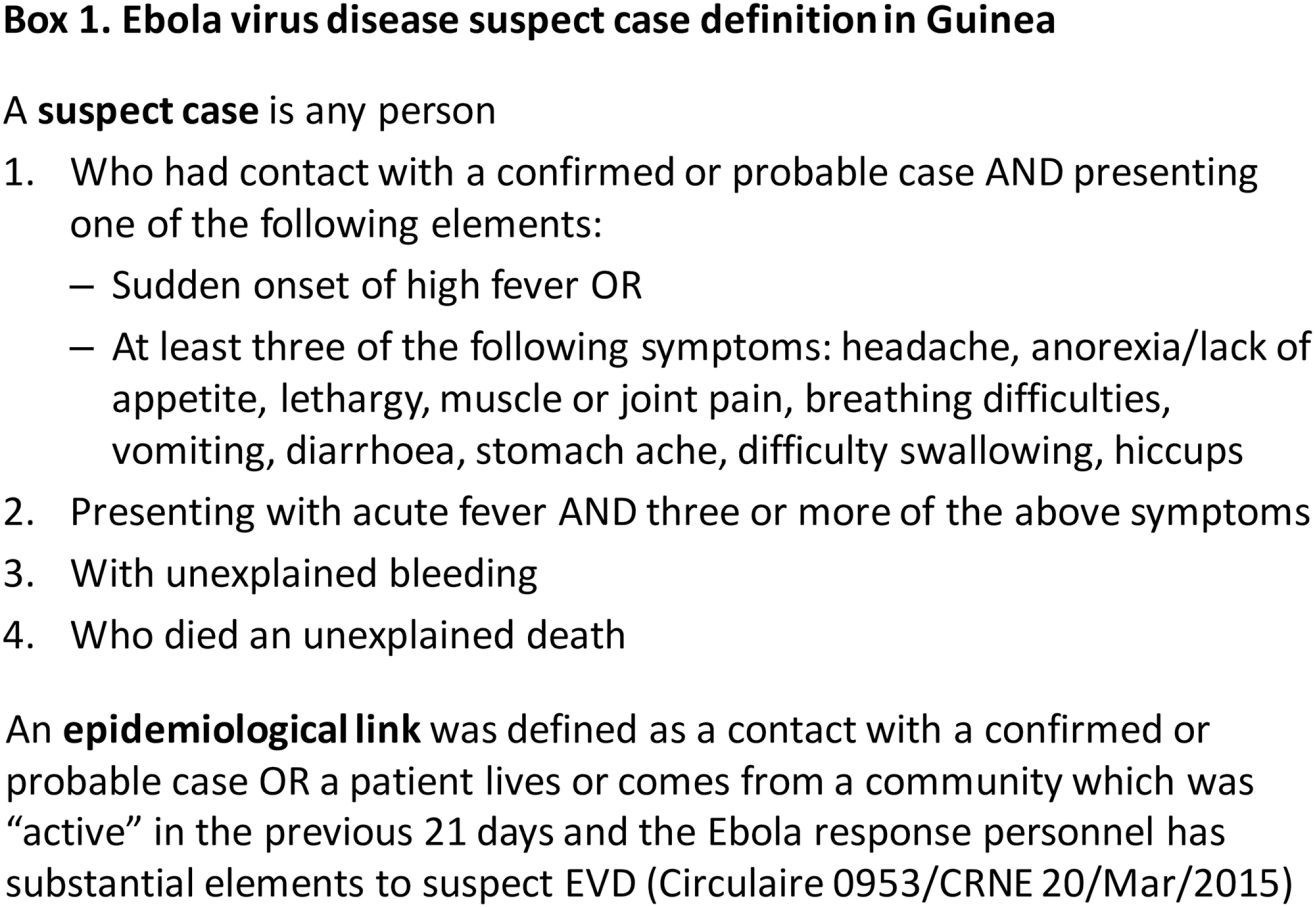
Ebola virus disease suspect case definition in Guinea [2].

During the 2014/15 Ebola Virus Disease (EVD) outbreak in West Africa Ebola Treatment Centres (ETC) functioned not only as isolation and care unit for confirmed EVD patients, but also as triage point for any ill person possibly suffering from EVD. After anamnestic screening, patients meeting the EVD suspect case definition established by the Guinean Ministry of Health and World Health Organization (see Fig 1) were admitted to isolation wards for suspect patients, whilst waiting for definitive diagnosis by confirmatory EVD testing relying on reverse-transcriptase polymerase chain reaction (EBOV RT-PCR) [1]. Upon result, confirmed cases were moved to separate isolation wards and non-cases (PCR negatives) discharged.

In Guinea, the Conakry ETC was the main referral centre for the capital region with an estimated population of three million people. Between March 2014 and November 2015 when the last case was discharged, 2565 EVD suspects were admitted and tested by EBOV RT-PCR. In Conakry, even though secondary and tertiary health care facilities have continued to consult and admit patients throughout the outbreak, difficulties in determining the risk of exposure to EVD have resulted in delays in access to appropriate health care [3]. Even after having tested negative for EVD, general health care facilities were not always willing to admit patients due to the potential risk of nosocomial infection.

The characteristics and case fatality of EVD confirmed cases have been widely described [4,5]. On the contrary, for non-cases published literature is scarce and limited to the diagnostic performance of the EVD suspect case definition. Outcomes and risk factors associated with death of non-cases have not been studied before. The proportion of non-cases among EVD suspects is generally important though, as the case definition for EVD is very broad and includes symptoms common for a long list of possible differential diagnoses. In ETCs in Freetown and Kailahun, Sierra Leone and in Bong County, Liberia, 36%, 33% and 58% out of 850, 419 and 382 admissions respectively were PCR negative, and thus non-cases [6–8].

Beyond case- and workload, admission of non-cases in an ETC prompts also outcome-related concerns. Non-cases risk contracting a nosocomial EVD infection in the ETC suspect wards, where EVD-positive and negative individuals would be mixed in the same ward while waiting for PCR results. This remained a major concern throughout the outbreak, but so far no such nosocomial infections have been confirmed, even after investigation of patients who were readmitted after first having tested negative for EVD [6]. In addition, an ETC stay may result, because of the limited diagnostic capacity and obligatory barrier care, in sub-standard care for non-cases with another urgently treatable disease.

Using routinely collected data of all suspect EVD patients admitted to the Conakry ETC in Guinea, we aimed to 1) describe the burden of non-cases in relation to the phase of the outbreak; 2) determine the duration of their stay at the ETC and (potential) subsequent nosocomial infections; and 3) compare characteristics, outcome and risk factors for death in confirmed cases and non-cases, in order to improve the selection, diagnosis and/or care of EVD suspects.

## Methods

### Study design and setting

Towards the end of the 2014/15 EVD outbreak in Guinea, we conducted a retrospective cohort study of all EVD suspects admitted to the ETC in Conakry between the first admission in the ETC on March 25^th^ 2014, and September 14^th^ 2015. The Conakry ETC was managed by Médecins sans Frontières and for most of the outbreak located within the Donka University Hospital, the largest health care facility in the country. In July 2015 the ETC was moved to a semi-permanent facility in another area of Conakry, Nongo.

### Patient flow with diagnostic procedures

Patients presenting at the ETC triage were referred either from other health facilities, through follow up of contacts of known EVD cases, or presented spontaneously (self-referral). Upon arrival, patients were screened by history against the EVD suspect case definition (see Fig 1) by trained clinicians. If a patient did not meet the case definition, s/he was not admitted and referred to a general health facility or discharged home. If the case definition was met, the patient was admitted to the isolation ward for EVD suspect cases where a venous blood sample for confirmatory testing was taken and standard supportive care (antimalarial drugs, antibiotics) started.

EVD infection was confirmed using a quantitative RT-PCR assay to detect viral RNA. Between March 2014 and July 2015 confirmatory testing was carried out by the National Laboratory of Viral Haemorrhagic Fever at Donka University Hospital using Taqman RT-PCR assays on whole blood samples which run 40 cycles (i.e. reaching a Cycle threshold value of 40) [4]. Results were available at a median of 5.6 hours (IQR 4.9–7.0) after blood sampling, which was done three times a day [9]. Between January 28 and February 10 2015, for at least 43 patients, heparin instead of EDTA tubes have mistakenly been used when drawing blood [10]. From May 2015 onwards the Xpert Ebola Assay (Cepheid GeneXpert Instrument Systems) was used, initially in parallel for validation and later as standard test to confirm EVD. The GeneXpert was operated in a laboratory within the ETC compound and blood sampling was no longer limited to three times a day, but performed upon arrival of the patient. GeneXpert testing allowed more rapid clinical decision making with results obtained within a median 2.7 hours (IQR 2.5 to 3.3 hours) after blood sampling [9].

If the RT-PCR test was positive, i.e. viral RNA was detected, the patient was transferred to an isolation ward for confirmed EVD cases. Patients who tested negative were discharged from the ETC, unless symptoms had started less than 72 hours prior to admission. For the latter, EVD was only ruled out after repeat PCR testing 72 hours after symptom onset. We use the term ‘non-cases’ for patients who were admitted as suspect cases in the ETC, but for whom EVD was definitively ruled out by diagnostic PCR.

Non-cases, alive at discharge, were sent home or transferred to a regular health care facility. Though guidelines foresaw the active follow-up of the discharged as EVD contact during the incubation period of a possible EVD infection contracted during his/her stay in the isolation ward, this was not always possible due to capacity constraints of the contact tracing teams. Follow-up on outcome of non-cases was limited to the time spent in the ETC while waiting for a definitive negative diagnostic EBOV-PCR. Data on deaths among non-cases which occurred after discharge from the ETC for the same illness episode were not available. For confirmed cases the outcome was documented for the entire course of illness (up to death or cure/discharge).

In addition to EVD diagnostic testing, the ETC laboratory also carried out Malaria rapid diagnostic tests (SD BIOLINE Malaria Ag P.f, Standard Diagnostics Inc.).

### Data collection and analysis

Data were combined from routine case notification forms, patient medical files and laboratory results for all patients admitted during the study period. At triage, standardised notification forms were filled in by trained clinicians, recording history, symptoms upon admission and demographic characteristics. The date of symptom onset, type of referral, the outcome at discharge and the date of discharge were retrieved from copies of the medical files held outside the isolation ward. Outcomes at discharge from the ETC included death, discharged home (i.e. cured for confirmed cases), or referral. The outcome was unknown when a patient decided to leave the ETC before being discharged. For ten cases referred to another ETC (exclusively for health care staff) outcomes at discharge of the other ETC were added for the analysis. Referral of non-cases took place only after EVD infection had been excluded as described above.

Clinical and demographic characteristics and outcome at ETC discharge are reported as frequencies or medians with range and interquartile range. Differences in dichotomous variables between cases and non-cases were analysed using Pearson’s Chi-squared test or Fisher’s exact test (when less than five cases or non-cases presented the sign). Mann-Whitney U test was used for differences in age and in delay of admission. Sensitivity, specificity, positive and negative predictive value, crude positive and negative likelihood ratios were computed for every symptom or sign at admission. Risk factors for deaths were computed through bivariate and multivariate analysis using unconditional logistic regression in the form of odds ratios (OR). All variables tested in the multivariate model were categorized: age, sex, current residence, type of referral, case load in the ETC (below 50, or 50 or more ETC admissions in the week a patient is admitted), increases in case load (below 20, or 20 or more extra admissions as compared to the previous week) and the delay of admission. 95% confidence intervals and p-values were computed using Likelihood ratio tests. Statistical analyses were performed using R and Stata 12 [StataCorp. College Station, TX].

Only routinely collected programme data were collected, anonymized, and analysed. The Ebola intervention and Conakry ETC were a joint project of the Ministry of Health of Guinea and Médecins Sans Frontières. The study fulfilled the exemption criteria set by the Ethics Review Board (ERB) of Médecins Sans Frontières (MSF), Geneva, Switzerland.

## Results

### Admission rates and length of stay for cases and non-cases

**Fig 2:**
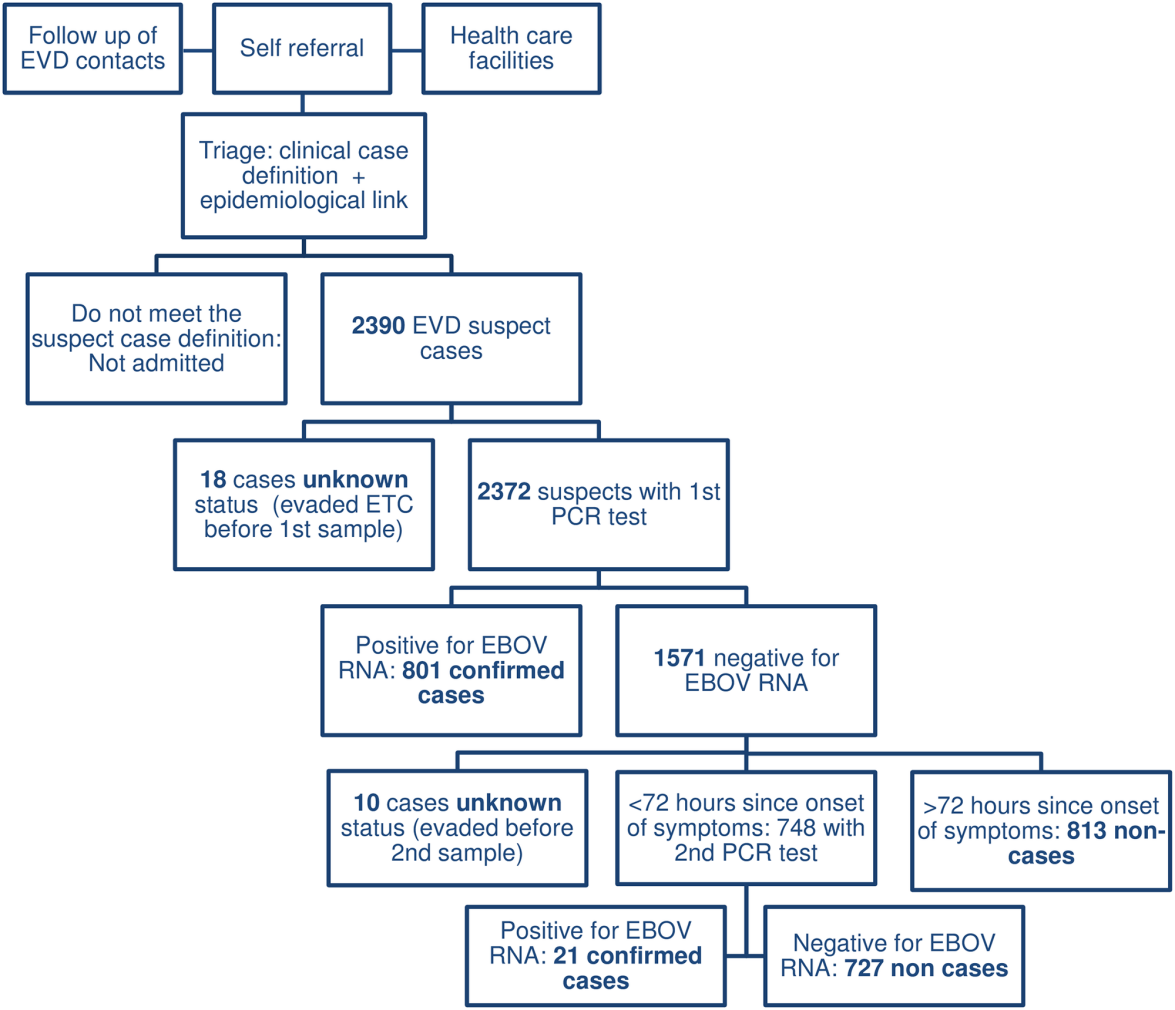
Case classification for suspect cases admitted to the Conakry ETC between March 25 2014 and September 14 2015.

Between 25 March 2014 and 14 September 2015, 2390 individuals were admitted as suspect EVD cases to the Conakry ETC. 2372 admitted patients underwent confirmatory testing. 822 (34.8%) were diagnosed with EVD, either after a single RNA positive EBOV RT-PCR test (n = 801) or after a second test at least 72 hours after symptom onset (n = 21). 1540 (65.2%) admitted suspects tested negative by RT-PCR and were designated as non-cases, following single (n = 813) or repeated (n = 727) negative RT-PCR. 18 patients chose to leave the ETC before any confirmatory testing and 10 underwent an initial PCR but evaded before a second test could confirm a diagnosis (Fig 2). Also 31 dead bodies of patients who died in the community or during referral to the ETC (including 7 EVD positive) were disposed at the ETC.

**Fig 3:**
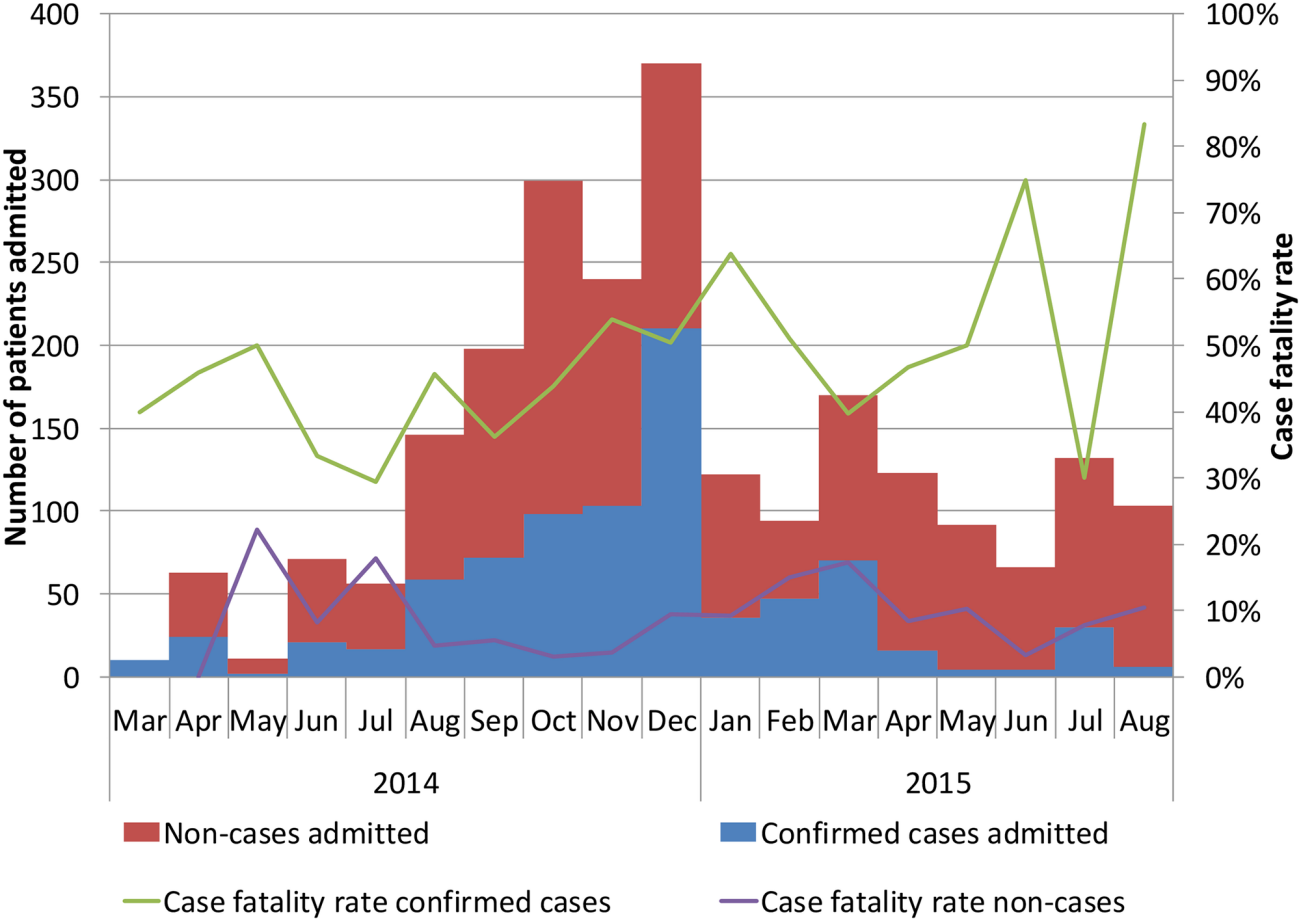
Frequency of admissions to the Conakry ETC and case fatality rates of EVD confirmed and non-cases each month between March 2014 and August 2015. Cases are classified according to outcome of confirmatory testing: cases confirmed by Ebola PCR, non-cases testing negative on EBOV PCR and cases with unknown status that left the ETC before being tested.

The largest number of admissions to the ETC was seen in the last weeks of December 2014, with a peak of 92 admissions in epidemiological week 51, including 52 EVD confirmed cases, 39 non-cases and one unknown case. During the study period, a median of 9 confirmed cases (IQR 2–18) and 19 non-cases (IQR 13–29) were admitted per week. Admissions of confirmed cases outnumbered those of non-cases only at the start of the outbreak in March 2014 and when case-loads were highest in December 2014 (Fig 3). Over the outbreak, the number of admissions of non-cases has remained steadier than that of cases, mounting up to 10 or more non-cases admitted and tested each week, even in weeks with few or no confirmed cases.

31.7% (748/2362) of all EVD suspects or 47.2% (727/1540) of the non-cases underwent repeated diagnostic RT-PCR testing and therefore stayed longer than one day in the suspect isolation ward. Among the 822 confirmed EVD cases, 21 only got confirmed after that second RT-PCR test, thus yielding false negative initial PCR results. However, 14 of the 21 false negative first PCR tests occurred between January 28 and February 10 2015, when wrong sampling tubes (heparin instead of EDTA) were used. The remaining 7 false negative initial PCR tests all occurred before the incident. Two of those during one day in the beginning of the outbreak, and three occurred in the week with the highest caseload in December 2014. No false negative initial PCR results occurred after February 2015, on a total of 154 confirmed cases and thus none after introducing the Xpert Ebola Assay in May 2015, on 45 confirmed cases. Non-cases who needed only one PCR stayed on average 0.80 days (median 1; IQR 0–1; range 0–3), but those who needed a second PCR test to exclude EVD had to stay on average 2.49 days (median 2; IQR 2–3; range 1–5).

Four (initial) non-cases were readmitted. Three of them were also PCR negative at the second admission as suspect case. One patient who had tested PCR negative in the weeks with the tube incident, was readmitted a week after leaving the ETC, tested positive and died 6 days later.

### Characteristics of confirmed EVD cases and non-cases

The age distribution among cases and non-cases was similar, though there were slightly more young children among the non-cases. Significantly more non-cases were male (61.7% vs 51.7%, p<0.001). The proportion residing in the capital region Conakry at the time of admission was higher for non-cases (67.9%) than for cases (54.9%, p<0.001).

The median delay between onset of symptoms and admission was similar. The longest delay recorded between onset of symptoms and admission for a case was 29 days, whereas a delay before admission of more than a month occurred in 6 non-cases.

Generalised fatigue and fever were the most common symptoms upon admission in confirmed and non-cases. Nausea or vomiting, diarrhoea, fatigue, loss of appetite, swallowing problems, joint aches, hiccups, unexplained bleeding and ocular redness/bleeding were less frequent among non-cases. Abdominal pain, headache, breathing problems, unexplained bleeding other than conjunctival bleeding, sore throat and coma were more frequent among non-cases (Table 1).

**Table 1:** Characteristics of confirmed EVD cases and non-cases upon admission.

	Confirmed cases (%)	Non-cases (%)	p- value
**Median age in years (IQR**, range**)**	30.0 (IQR 20–41, range 0–87)	28.0 (IQR 19–40, range 0–96)	**0.389**
**Age group**			
**1–4 years of age**	43 (5.2%)	113 (7.4%)	**0.031**
**5–18 years**	112 (13.6%)	200 (13.1%)	0.913
**18–49 years**	543 (66.1%)	956 (62.6%)	ref
**50 or more years**	123 (15.0%)	258 (16.9%)	0.151
**Male sex**	51.7%	61.7%%	**<0.001**
**Delay of admission (number of days between symptoms onset and admission), median (IQR, range)**	4 (IQR 2–6, range 0–29)	3 (IQR 1–5, range 0–61)	**0.691**
**Clinical signs**			
**Fever**	585 (71.5%)	1060 (69.5%)	0.300
**Nausea/Vomiting**	391 (47.8%)	640 (42.0%)	**0.007**
**Diarrhoea**	292 (35.7%)	423 (27.7%)	**<0.001**
**Fatigue**	693 (84.7%)	1168 (76.6%)	**<0.001**
**Loss of appetite**	515 (63.0%)	855 (56.1%)	**0.001**
**Abdominal pain**	231 (28.2%)	526 (34.5%)	**0.002**
**Thoracic pain**	61 (7.5%)	115 (7.5%)	0.942
**Muscle pain**	268 (32.8%)	441 (28.9%)	0.053
**Joint ache**	330 (40.3%)	528 (34.6%)	**0.006**
**Headache**	407 (49.8%)	835 (54.8%)	**0.021**
**Cough**	87 (10.6%)	201 (13.2%)	0.074
**Breathing problems**	21 (2.6%)	84 (5.5%)	**0.001**
**Swallowing problems**	89 (10.9%)	91 (6.0%)	**<0.001**
**Sore throat**^a^	4 (0.5%)	24 (1.6%)	**0.026**
**Hiccups**	84 (10.3%)	110 (7.2%)	**0.011**
**Unexplained bleeding**	206 (25.2%)	325 (21.3%)	**0.033**
** - *Ocular redness/bleeding***^b^	*170 (20*.*8%)*	*130 (8*.*5%)*	***<0*.*001***
** - *Other***	*62 (7*.*6%)*	*229 (15%)*	***<0*.*001***
**Coma**	7 (0.9%)	58 (3.9%)	**<0.001**
**Skin redness**^a^	15 (1.0%)	3 (0.4%)	0.137
**Photosensitivity or ocular pain**^a^	0 (0.0%)	2 (0.1%)	0.546
**Confusion or disorientation**^a^	2 (0.3%)	15 (1%)	0.070
**Jaundice**	0 (0.0%)	0 (0.0%)	
**Type of referral**^c^	n = 25156 (22.3%)	n = 620228 (36.8%)	**<0.001**
**ETC ambulance**	57 (22.7%)	116 (18.7%)	
**Self-referral**	78 (31.1%)	145 (23.4%)	
**University hospital**	1 (0.4%)	27 (4.4%)	
**Not recorded**	59 (23.5%)	102 (16.5%)	
**Current residence**			**<0.001**
**Conakry (capital)**	451 (54.9%)	1045 (67.9%)	
**Outside Conakry**	371 (45.1%)	486 (31.6%)	
**Health care worker**	99 (12.0%)	106 (6.9%)	**<0.001**

IQR, Interquartile range

^a^ Fisher’s exact test was used to compute the p-values when the n below five among either cases or non-cases.

^b^ Ocular redness/bleeding refers to conjunctivitis and conjunctival bleeding. Data were not recorded separately.

^c^ The type of referral was recorded from December 18 2014 onwards and in the analysis of referral type only admitted suspect cases from within Conakry were considered

Table 2 shows crude positive and negative likelihood ratios for EVD confirmation of the clinical signs. Only suspects with ocular redness/bleeding were more than twice more likely to be cases than to be non-cases (positive likelihood ratio of 2.44). Ocular redness/bleeding was present among 20.8% of confirmed cases. Suspects with breathing problems, sore throats, coma, skin redness and confusion or disorientation were more than twice more likely to be non-cases than to be cases (positive likelihood ratio below 0.5). These latter symptoms were rare though, all present in less than 6% of non-cases. The negative likelihood ratios did not yield differences of a factor 2.

**Table 2:** Clinical predictors of EVD confirmation when admitted to the Conakry ETC: Sensitivity, specificity, positive and negative predictive values and positive and negative likelihood ratios.

Clinical signs	Sensitivity	Specificity	Positive Predictive value	Negative Predictive value	Likelihood Ratio Positive	Likelihood Ratio Negative
**Fever**	71.5%	30.5%	35.6%	66.7%	1.03	0.93
**Nausea/Vomiting**	47.8%	58%	37.9%	67.5%	1.14	0.90
**Diarrhoea**	35.7%	72.3%	40.8%	67.7%	1.29	0.89
**Fatigue**	84.7%	23.4%	37.2%	74.1%	1.11	0.65
**Loss of appetite**	63.0%	43.9%	37.6%	68.9%	1.12	0.84
**Abdominal pain**	28.2%	65.5%	30.5%	63.0%	.82	1.10
**Thoracic pain**	7.46%	92.5%	34.7%	65.1%	.99	1.00
**Muscle pain**	32.8%	71.1%	37.8%	66.3%	1.13	0.95
**Joint ache**	40.3%	65.4%	38.5%	67.1%	1.17	0.91
**Headache**	49.8%	45.2%	32.8%	62.7%	.909	1.11
**Cough**	10.6%	86.8%	30.2%	64.4%	.807	1.03
**Breathing problems**	2.6%	94.5%	20%	64.4%	**.466**	1.03
**Swallowing problems**	10.9%	94.0%	49.4%	66.3%	1.82	0.948
**Sore throat**	0.5%	98.4%	14.3%	64.8%	**.311**	1.01
**Hiccups**	10.3%	92.8%	43.3%	65.8%	1.42	0.97
**Unexplained bleeding**	25.2%	78.7%	38.8%	66.2%	1.18	0.95
**Ocular redness/bleeding**^a^	20.8%	91.5%	56.7%	68.3%	**2.44**	0.87
**Coma**	0.9%	96.1%	10.8%	64.5%	**.226**	1.03
**Skin redness**	0.4%	99.0%	16.7%	65.0%	**.374**	1.01
**Photosensitivity or ocular pain**	0%	99.9%	0	65.1%	0	1.00
**Confusion or disorientation**	0.2%	99.0%	11.8%	65%	**.249**	1.01
**Clinical criteria suspect case definition**^b^	56.9%	46.4%	36.3%	66.8%	1.06	0.93
**Three major signs**^c^	27.7%	79.1%	41.5%	67.2%	1.33	0.91

^a^ Ocular redness/bleeding refers to conjunctivitis and conjunctival bleeding

^b^ The suspect case definition in Guinea’s clinical criteria (when no epidemiological link can be established) are “Any person presenting with acute fever AND presenting three or more of the following: headache, anorexia/lack of appetite, lethargy, muscle or joint pain, breathing difficulties, vomiting, diarrhoea, stomach ache, difficulty swallowing, hiccups” or “Any person with unexplained bleeding”.

^c^ Presenting with three major signs as identified by Lado et al [8]: intense fatigue, confusion, conjunctivitis, hiccups, diarrhea, or vomiting.

The clinical criteria of the suspect EVD case definition, thus not considering contact history (i.e acute fever and presenting three or more other specific signs, Fig 1) were met in 56.9% of cases and 53.6% of non-cases.

### Mortality and associated risk factors among cases and non-cases

Among 822 confirmed cases and 1540 non-cases respectively 377 (45.9%) and 98 (6.4%) died during their stay in the ETC, thus non-cases accounted for 20.6% (98/475) of all deaths in the ETC. The median length of stay in the ETC between admission and death was 4 days (IQR 2–6, range 0–17) for confirmed cases. Most (58/98, 59.2%) non-cases died on the day of admission, 33 during the 2^nd^ day in the ETC suspect ward and the remaining four on the 3^rd^ or 4^th^ day. After testing negative for EBOV PCR, 256 non-cases were transferred for further health care and no outcome after discharge from health care is known (Table 3).

**Table 3:** Outcome at discharge of the ETC for all admitted patients in the ETC Conakry between 25 March 2014 and 14 September 2015.

Outcome	Confirmed case	Non-case	EVD PCR unknown	Total
**Patients who died before admission**	8	23	0	**31**
**Patients alive upon admission**	822	1540	28	**2390**
• ***Discharged***	*444 (54*.*0%)*	*1180 (76*.*7%)*	*0*	*1624 (67*.*9%)*
• ***Died***	*377 (45*.*9%)*	*98 (6*.*4%)*	*0*	*475 (19*.*9%)*
• ***Transferred***		*256 (16*.*6%)*	*0*	*256 (10*.*7%)*
• ***Unknown***	*1 (0*.*1%)*	*6 (0*.*4%)*	*28*	*35 (1*.*5%)*
**Total**	830	1563	28	**2421**

EVD, Ebola viral disease; PCR, Polymerase chain reaction

The monthly trend of case fatality rate of cases and of non-cases is plotted against the total number of admissions in the ETC in Fig 3.

Table 4 summarizes, for specific patient characteristics, the case fatality rate and the strength of association with fatal outcomes in the ETC, among cases and non-cases. For non-cases, residing in Conakry was identified as independent risk factor for a fatal outcome in the ETC (aOR 1.78 95%CI 1.08–2.96). Non-cases were less likely to die when admitted during a week with 20 or more extra admissions than the previous week (aOR = 0.31; 95%CI 0.17–0.58) or a week with 50 or more admissions (OR = 0.61; 95%CI 0.38–0.96). Differently from cases, dying among non-cases was not significantly associated with age below five or over fifty. No interactions or important confounding was observed between the risk factors for mortality we recorded.

**Table 4:** Bivariate and multivariate analysis of the association with possible predictors for higher mortality in confirmed EVD cases and non-cases. Patients who died before admission were excluded from the analysis.

Risk factor		Confirmed EVD cases			Non-cases		
		Case fatality rate	Crude OR of dying (95% CI)	Adjusted OR (95% CI)	Case fatality rate	Crude OR of dying (95% CI)	Adjusted OR (95% CI)
**Age group**	0 to 4 y	69.8%	**2.85 (1.45–5.58)**	**3.22 (1.62–6.38)**	2.7%	0.58 (0.21–1.63)	0.69 (0.24–1.96)
	5 to 17 y	24.1%	**0.39 (0.25–0.62)**	**0.39 (0.24–0.62)**	6.0%	1.09 (0.59–2.04)	1.13 (0.60–2.12)
	18 to 49 y	44.6%	ref	ref	5.5%	ref	ref
	50+ y	63.4%	**2.14 (1.42–3.21)**	**2.10 (1.39–3.17)**	9.3%	1.62 (0.98–2.66)	1.54 (0.93–2.56)
**Sex**	Female	40.7%	ref	ref	6.4%	ref	
	Male	50.8%	**1.50 (1.14–1.98)**	**1.62 (1.21–2.16)**	6.6%	1.04 (0.69–1.56)	
**Current residence**	Conakry	41.6%	**0.68 (0.52–0.90)**	**0.67 (0.50–0.94)**	7.1%	**1.72 (1.0–2.80)**	**1.78 (1.08–2.96)**
	Other region	51.2%	ref	ref	4.5%	ref	ref
**Case load/week**	50 or more admissions	46.7%	1.08 (0.82–1.43)		4.8%	**0.61 (0.38–0.96)**	
	Less than 50 admissions	44.0%	ref		7.1%	ref	
**Increase in case load with previous week**	Less than 20 extra admissions	46.3%	ref		7.8%	ref	ref
	20 or more extra admissions	45.0%	0.94 (0.70–1.26)		2.9%	**0.33 (0.18–0.59)**	**0.31 (0.17–0.58)**
**Delay of admission from symptom onset**	Less than 5 days	42.2%	ref	ref	6.1%	ref	
	5 or more days	50.4%	**1.38 (1.04–1.82)**	1.33 (0.99–1.79)	5.7%	0.90 (0.57–1.43)	
**Type of referral**	Non specified ambulance	50.0% (n = 56)	ref		4.0% (n = 225)	ref	
	ETC ambulance	38.6% (n = 57)	0.63 (0.30–1.32)		11.2% (n = 116)	**2.71 (1.15–6.39)**	
	Self-referral	42.3% (n = 78)	0.73 (0.37–1.46)		9.0% (n = 145)	2.12 (0.90–4.97)	
	University hospital	0.0% (n = 1)			3.7% (n = 27)	0.83 (0.10–6.72)	
	Not recorded	57.6% (n = 59)	1.36 (0.65–2.84)		17.6% (n = 102)	**5.24 (2.35–11.68)**	
**Health care worker**	Yes	44.4%	0.96 (0.63–1.47)		3.8%	0.53 (0.19–1.49)	
	No	45.3%	ref		6.5%	ref	

EVD, Ebola viral disease; OR, Odds ratio; 95% CI, 95% Confidence interval; ref, reference; y, year; ETC, Ebola Treatment Centre; In the multivariate analysis model of the confirmed cases, adjustments were made for age (groups), sex, the delay between onset of symptoms and admission, and the current residence of the patient. For the non-cases adjustments were made for age (groups), the current residence of the patient and for an increase in case load.

## Discussion

Little attention has been given to the non-cases, suspect cases that tested negative for EVD, despite serious challenges such as 1) the possibility of nosocomial EVD infection during their stay in the isolation ward, 2) additional workload in the ETC when a large number of non-cases also require care and blood sampling, 3) missed opportunities for emergency care for non-cases in need of intensive care for another condition, 4) the stressful experiences the concerned patients underwent during their stay in the ETC and 5) the difficult and delayed access to regular health care facilities when EVD can only be excluded in an ETC.

Our study highlights the importance of considering the non-cases when designing referral, diagnosis and care of EVD suspects. Almost two thirds of admitted EVD suspects were non-cases and one in five deaths occurring in the ETC was a non-case dying from another condition than EVD before RT-PCR results were available. Other ETCs have also reported high proportions of non-cases, ranging between 33 and 58% [6,8,11], although never as high as the 65% non-cases among admissions in Conakry. Our results show that about 30% of cases and non-cases are being admitted without fever, in part likely a result of the difficulty to accurately measure body temperature due to biosecurity measures at triage and in the isolation wards. The most frequent symptoms have small differences in frequency between confirmed and non-cases. Specific symptoms as ocular redness/bleeding, breathing difficulties, sore throat, coma, skin redness and confusion or disorientation yield positive likelihood ratios that could allow differentiating cases from non-cases but their rare occurrence limits their usefulness. From our data, it is unlikely that a combination of symptoms alone can yield sufficient sensitivity and specificity to replace the clinical signs as currently used. Other studies have proposed a combination of three or more specific signs [8], or using a prediction score combining risk factors and symptoms to determine which patients to admit to the ETC [7]. Applying the combination of three or more of the symptoms proposed by Lado et al on this cohort, would yield an even lower sensitivity of 27,7% but an improved specificity of 79.1%. Differentiating EVD based on the current clinical criteria is difficult but no viable changes to the EVD suspect case definition can be proposed from our data.

ETC inpatient mortality among non-cases in rural Liberian and Sierra Leonean ETCs was 5.4% and 4.7% respectively [7,11], both slightly lower than, but in range with the 6.4% we observed in the Conakry ETC. From the medical files we could not retrieve enough detail on diagnoses of non-cases who died within the ETC to conclude whether those deaths were avoidable. It is likely that the impact of speeding up diagnosis would be limited for moribund non-cases. Nevertheless, diagnostic specific treatment of non-cases was delayed due to the passage in the suspect isolation ward. As no follow up data after discharge or referral of non-cases were available, our data cannot provide the full impact on mortality of delaying treatment for certain conditions while waiting in the ETC for a negative EBOV-PCR.

In future EVD outbreaks, the current set-up requiring testing through RT-PCR after referral to a centralised isolation ward may continue to compromise diagnosis and care of non-cases and interfere and delay care of confirmed cases. Even with PCR confirmatory testing yielding results within 2 hours [9], hours to days are spent on patient transfer to an ETC and—for almost half of the non-cases, while waiting for a second PCR test in the isolation ward. Integrating point-of-care RT-PCR EVD testing as part of triage at the larger health care facilities, coupled with greater emphasis on keeping these general health facilities functional during outbreaks, and intensified health education to promote early care-seeking behaviour, would allow more rapid diagnosis and quicker access to appropriate care for any suspected EVD case.

We observed moreover that using the point-of-care Xpert RT-PCR Ebola Assay no EVD cases were missed when only a single confirmatory test would have been carried out. In the Conakry ETC most false negative initial PCR results, all before February 2015, were related to errors in the sampling tubes used. Of the remaining false negatives five out of seven occurred clustered over a few days, also possibly suggesting a quality problem. The Xpert RT-PCR Ebola Assay has been proven to be at least as performant as several common laboratory-based assays, with no false negative results reported in studies on whole blood samples [12–14]. Additional reviews of data from the other major ETCs would allow to assess whether the rule of a second confirmatory test at least 72 hours after onset of symptoms should be maintained in all circumstances.

A case report of a false negative test of an asymptomatic high risk contact in Monrovia in September 2014 [15] argued for repeat testing. However, being asymptomatic, this was an exceptional case which would also have been detected when initial symptoms appeared, through a well-functioning contact tracing and follow up system. Cases testing negative in our study were admitted suspect cases, therefore presenting symptoms.

The one third of non-cases who lived outside Conakry had better chances for survival than those from Conakry. We assumed that referrals of severely ill non-EVD patients from the largest health care facilities in Conakry had a higher chance of dying, but our hypothesis was not confirmed from the limited records on the type of referral of suspect cases. Non-cases referred from further districts were more often contacts of known EVD cases referred by surveillance teams, and therefore in better health than critically ill patients referred from health care facilities.

Previous studies of confirmed cases found that younger age (below 5 years) or older age (over 50 years) are determinants for higher mortality among confirmed EVD cases [4,5,16,17], which our study confirmed. We did not notice this increased mortality in the youngest non-cases, but found also an association—although weaker, between older age and death, likely due to more severe comorbid conditions other than EVD in referred elderly people. We also observed higher case fatality among male confirmed cases than among females, a difference we did not observe among non-cases. This is in contrast to what we would have expected, i.e. that pregnancies, a known risk factor among female cases, would in general have increased case fatality among females. Unfortunately since pregnancies were poorly recorded, we were not able to assess the effect pregnancy had on mortality.

The number of admissions of confirmed cases started to increase from August 2014, reaching its peak in December 2014 when bed capacity was fully reached and work load for the ETC staff was overwhelming. However, this was not mirrored by an increased case fatality rate among cases and non-cases during these weeks. Paradoxically a decreased case fatality among non-cases was seen in weeks with increasing admissions (from 7.9 to 2.9%). One would have expected that the extra work load could have a detrimental effect on case fatality during the busiest weeks or weeks with large increases in the number of admissions. Hypothetically, this might be due to severely ill non-cases with severe conditions being referred especially at times when the risk of nosocomial infection in the ETC is considered low, whereas in weeks with increased numbers of admissions proportionally more contacts of known EVD cases with acute illnesses are referred, and they have better chances of survival.

Although much feared, we found no cases of nosocomial EVD infection contracted while staying in the isolation ward for EVD confirmatory testing. Only one patient out of 1436 discharged or transferred non cases was readmitted and tested positive after the second admission. This patients’ initial negative result was false negative following an error with the blood sampling tubes.

There are a number of important limitations to this study. We had no final illness-episode outcomes available for 256 (16.6%) of the 1540 discharged non-cases, who were transferred to regular health facilities after EVD infection was excluded, which limits our case fatality findings. We could only analyse deaths up to the moment of transfer from the ETC, probably underestimating the total number of deaths among non-cases. More detail on diagnosis and causes of death of non-cases may have provided more insight on how and what proportion of deaths among non-cases could have been averted. Another limitation is that other possible drivers for the large proportion of non-cases could not be assessed: When the population started to gain confidence in the ETC, patients for whom no care options were available elsewhere may have started seeking care in the ETC; During times with higher epidemic intensity health care facilities demanded ill patients to first get tested in the ETC.

## Conclusions

Our findings on non-cases in Conakry, studied during most of the West African Ebola outbreak, highlight the importance of considering non-cases when setting up triage and referral of EVD suspect cases. Centralising triage at the ETCs comes at a cost: the majority of admissions are non-cases in need of treatment for other conditions, and even though non-cases were only admitted for a maximum of three days until EVD confirmation, mortality may have been different when these patients would have been immediately admitted to a hospital where stabilization and critical care, as well as diagnose-specific examinations and treatment can be practiced without the limits of full-barrier care.

No combination of symptoms with sufficient sensitivity and specificity to differentiate EVD cases from non-cases can be proposed from our data. Other options to consider to overcome delays in access to adapted care for cases and non-cases are 1) the integration of highly sensitive EVD diagnostic tests with short turnaround time in the triage at peripheral hospitals, or 2) speeding up the diagnostic timeframe by dropping the second confirmatory EVD test for those presenting at the ETC and health care facilities less than 72 hours after symptom onset. This latter strategy should be backed up by a sound contact tracing and follow-up system. Both strategies need further research to ensure feasibility and that outbreak control is not compromised.
